# Pharmacokinetics of thiotepa in high-dose regimens for autologous hematopoietic stem cell transplant in Japanese patients with pediatric tumors or adult lymphoma

**DOI:** 10.1007/s00280-019-03914-2

**Published:** 2019-08-19

**Authors:** Eisei Kondo, Takashi Ikeda, Hiroaki Goto, Momoko Nishikori, Naoko Maeda, Kimikazu Matsumoto, Hideo Kitagawa, Naoto Noda, Saori Sugimoto, Junichi Hara

**Affiliations:** 1 Department of Hematology and Oncology, Okayama University Graduate School of Medicine, Dentistry and Pharmaceutical Sciences, Okayama, Japan; 2 Department of Hematology, Kawasaki Medical School, 577 Matsushima, Kurashiki, 701-0192 Japan; 3 Division of Hematology and Stem Cell Transplantation, Shizuoka Cancer Center, Shizuoka, Japan; 4 Hematology/Oncology and Regenerative Medicine, Kanagawa Children’s Medical Center, Yokohama, Japan; 5 Department of Hematology and Oncology, Graduate School of Medicine, Kyoto University, Kyoto, Japan; 6 Department of Pediatrics, National Hospital Organization Nagoya Medical Center, Nagoya, Japan; 7 Children’s Cancer Center, National Center for Child Health and Development, Tokyo, Japan; 8 Sumitomo Dainippon Pharma Co., Ltd., 6-8, Doshomachi 2-chome, Chuo-ku, Osaka, Japan; 9 Department of Pediatric Hematology/Oncology, Children’s Medical Center, Osaka City General Hospital, Osaka, Japan

**Keywords:** HSCT, Pharmacokinetics, TEPA, Thiotepa

## Abstract

**Purpose:**

Thiotepa is used in high-dose chemotherapy (HDT) before autologous hematopoietic stem cell transplantation (HSCT) to treat solid tumors and hematological malignancies. This Phase 1 study was conducted to establish the pharmacokinetics (PK) of thiotepa in a Japanese population.

**Methods:**

HDT/HSCT was performed in pediatric patients (≥ 2 years) with solid tumors or brain tumors (thiotepa 200 mg/m^2^/day IV-infused over 24 h on HSCT Days − 12, − 11, − 5, and − 4 and melphalan 70 mg/m^2^/day IV-infused over 1 h on Days − 11, − 5, and − 4) and adult patients (≥ 16 years) with malignant lymphoma (thiotepa 200 mg/m^2^/day 2-h IV-infusion on HSCT Days − 4 and − 3 plus busulfan 0.8 mg/kg 2-h IV-infusion every 6 h from HSCT Days − 8 to − 5). Pharmacokinetics of thiotepa were assessed following initial dose. Safety and efficacy were also evaluated.

**Results:**

Nine pediatric and 10 adult patients were enrolled. Mean volume of distribution (*V*_z_) of thiotepa normalized with body surface area (BSA) was lower for pediatric patients (16.4 L/m^2^) compared with adult patients (26.4 L/m^2^) as expected due to the higher specific surface area of children. Clearance and biological half-life were similar between pediatric and adult patients. Two serious adverse events (cardiac arrest and pulmonary edema) were observed. Survival rate (Day 100 post-HSCT) was 77.8% (95% CI 36.5–93.9%) for pediatric patients and 100% for adult patients.

**Conclusion:**

Thiotepa elimination was comparable in pediatric and adult patients with cancer. Lower *V*_z_ in pediatric compared with adult patients was expected. HDT with thiotepa prior to autologous HSCT was well tolerated.

**Study registration:**

Japic CTI-163433.

**Electronic supplementary material:**

The online version of this article (10.1007/s00280-019-03914-2) contains supplementary material, which is available to authorized users.

## Introduction

Thiotepa (*N*,*N*′,*N*″-triethylenethiophosphoramide) is an alkylating agent that has been used to treat solid tumors and hematological diseases since the 1950s [1, 2]. In vitro studies suggest that thiotepa is metabolized by cytochrome P450 3A4 (CYP3A4) and cytochrome P450 2B6 (CYP2B6) to triethylene phosphoramide (TEPA) [3], which shows a comparable alkylating activity to the parent drug [4]. Because of its broad-spectrum antitumor activity and relative lack of extramedullary toxicity, thiotepa has been incorporated in high-dose chemotherapy (HDT) with autologous hematopoietic stem cell transplantation (HSCT) for various solid tumors and hematological malignancies [5–9].

Both thiotepa and TEPA efficiently cross the blood–brain barrier, with cerebrospinal fluid levels in excess of 90% of serum levels [10]. As such, thiotepa in combination with carmustine or busulfan has been used to treat lymphoma with central nervous system (CNS) involvement as consolidative therapy in the high-dose setting [5, 11, 12]. In a randomized Phase 2 study (IELSG-32) of consolidative therapies in patients with primary CNS lymphoma, HDT with thiotepa and carmustine followed by HSCT showed comparable efficacy with no increase in cognitive impairment compared with whole-brain irradiation [6]. Two randomized Phase 2 studies (PRECIS [NCT00863460] and CALGB51101 [NCT01511562]), a non-randomized, single-group, open-label study [NCT01505569], and a Phase 3 study (MATRix [NCT02531841]) of thiotepa-based HDT/HSCT in primary CNS lymphoma and high-risk or relapsed solid tumors are ongoing [13–16].

Thiotepa is approved by the European Medicines Agency in adults and children, in combination with other chemotherapeutic agents, as both autologous and allogeneic HSCT therapy in hematological diseases and solid tumors [17]. In Japan, thiotepa has been approved for standard-dose chemotherapy since 1958, but not for HDT, and it is currently unavailable after manufacturing was discontinued in 2008. [18] There was also a supply shortage of thiotepa in the USA in 2014 [19], during which time the US Food and Drug Administration allowed temporary importation of thiotepa from an European supplier. As thiotepa was not approved in HDT/HSCT in Japan, we conducted this Phase 1 study (Japic CTI-163433) to establish the pharmacokinetics (PK) of thiotepa as a conditioning treatment for HDT/HSCT in a Japanese population. Safety and efficacy endpoints were examined as secondary objectives.

## Patients and methods

### Study design and treatment

This was an open-label, non-comparative study to investigate the PK of intravenous (IV) thiotepa (Sumitomo Dainippon Pharma [Osaka, Japan]) as a conditioning treatment for autologous HSCT in pediatric patients with solid tumors or brain tumors (with concomitant melphalan [Aspen Japan, Tokyo, Japan]) or adult patients with malignant lymphoma (with concomitant busulfan [Otsuka Pharmaceutical, Tokyo, Japan]). The study was conducted at seven clinical sites in Japan.

The study comprised a screening period, an 8- to 12-day conditioning period, a 28-day transplant period, and an outcome investigation period 100 days post-HSCT (Supplementary Fig. S1). The day of HSCT was defined as HSCT Day 0.

The study protocol and informed consent form were approved by the Institutional Review Board at each clinical site prior to study initiation. The study was conducted in accordance with the Declaration of Helsinki, the Pharmaceutical and Medical Devices Act, and Good Clinical Practice ordinance. All patients and/or their legal representatives provided written informed consent prior to participation in the study.

### Patients

Pediatric male and female patients ≥ 2 years of age with solid tumors or brain tumors or adult patients ≥ 16 years of age with malignant lymphoma, and who had hematopoietic cells collected for autologous HSCT (CD34-positive cells 2 × 10^6^/kg or nuclear cells 1 × 10^8^/kg), were eligible for inclusion in the study. Patients ≥ 16 years of age needed to provide voluntary written, informed consent. For patients 16–19 years of age, written informed consent was also required from the patient’s legal representative and for those < 16 years of age by the patient’s legal representative. For patients who were unable to provide consent (e.g. due to cognitive impairment), written informed consent was provided by the patient’s legal representative. Other inclusion criteria, which were to be met within 15 days prior to study registration were: Eastern Cooperative Oncology Group (ECOG) performance status 0–2; aspartate aminotransferase and alanine aminotransferase ≤ threefold, γ-glutamyltransferase ≤ 2.5-fold and total bilirubin and creatinine ≤ 1.5-fold the reference range at the clinical site; left ventricular ejection fraction ≥ 50%; and estimated glomerular filtration rate (eGFR) ≥ 60 mL/min/1.73 m^2^ for patients ≥ 18 years of age and ≥ 100 mL/min/1.73 m^2^ for patients < 18 years of age. For > 12 years old, eGFR = 194 × serum creatinine (mg/dL) −1.094 × age − 0.287 (× 0.739 for female). For < 12 years old, eGFR = 0.35 × [height (m)/serum creatinine (mg/dL)] × 100 were used to calculate eGFR.

Exclusion criteria included other treatment for the management of underlying disease within 20 days prior to treatment initiation; live vaccine received within 90 days prior to treatment initiation date; history of complications that may affect metabolism or excretion of drugs; active infection; serious hypersensitivity to thiotepa or polyethylene glycol 400, melphalan (for pediatric patients with solid tumors or brain tumors), or busulfan (for adult patients with malignant lymphoma); positive test for hepatitis B surface antigen or antibody, hepatitis B core antibody, hepatitis C virus antibody, or HIV (patients positive only for hepatitis B surface antibody could be enrolled if vaccinated for type B hepatitis); cardiac effusion, pleural effusion, or ascites requiring treatment; and consumption of grapefruit-containing foods or St. John’s wort within 13 days prior to treatment. Due to the occurrence of two serious adverse events (AEs; cardiac arrest and pulmonary edema) in patients who had undergone HSCT within the past 6 months, the following exclusion criteria were added after the study commenced: previous HSCT within 6 months prior to the study HSCT; difficulty receiving sufficient fluid replacement and frequent blood transfusion when concomitantly receiving melphalan; and cardiac effusion, pleural effusion, or ascites for which treatment had been received within 15 days prior to study registration.

### Treatment

#### Pediatric patients with solid tumors or brain tumors

Thiotepa 200 mg/m^2^/day was IV-infused over 24 h on HSCT Days − 12, − 11, − 5, and − 4. Melphalan 70 mg/m^2^/day was infused over 1 h via a separate IV line on HSCT Days − 11, − 5, and − 4. Thiotepa and melphalan were suspended if eGFR was < 45 mL/min/1.73 m^2^ for patients ≥ 18 years of age or < 75 mL/min/1.73 m^2^ for patients < 18 years of age on HSCT Days − 7, − 5, or − 4. Additionally, thiotepa and/or melphalan could be suspended or postponed, or the dose of melphalan reduced, based on the investigator’s judgement of the patient’s condition, with the subsequent HSCT and evaluation schedule adjusted accordingly.

#### Adult malignant lymphoma

Thiotepa 200 mg/m^2^/day was IV administered over 2 h on HSCT Days − 4 and − 3. Busulfan was IV administered at a dose of 0.8 mg/kg as 2-h infusion every 6 h (total dose 3.2 mg/kg/day) for 4 consecutive days from HSCT Days − 8 to − 5. Busulfan infusion could be suspended or postponed or the dose reduced, based on the investigator’s judgment of the patient’s condition, with the subsequent HSCT date and evaluation schedule adjusted accordingly. Since busulfan may induce convulsion, prophylactic use of an anti-convulsant was recommended (administered up to 48 h pre- and post-busulfan dose); levetiracetam (IV or PO) for pediatric patients and valproate sodium (PO) for adults.

### Assessments

#### Pharmacokinetic assessment

For an overview of PK sampling times, see Supplementary Fig. 1. For pediatric patients with solid tumors or brain tumors, blood sampling for PK assessment was conducted within 2 h prior to the HSCT Day − 12 thiotepa dose, immediately post-dose and 0.5, 1, 2, 4, and 6 h post-dose; note that the HSCT Day − 11 thiotepa and melphalan doses were initiated following completion of PK blood sampling on HSCT Day − 11. Samples were also collected within 2 h prior to the HSCT Day − 5 thiotepa dose and immediately following the HSCT Day − 4 dose. For adult patients with malignant lymphoma, blood samples were collected within 2 h prior to the HSCT Day − 4 thiotepa dose, immediately post-dose and 0.5, 1, 2, 4, 6, and 8 h post-dose, and within 2 h prior to, and immediately following, the HSCT Day − 3 thiotepa dose.

Blood volumes of 1 mL were collected at each time point and immediately centrifuged [room temperature, 3000 rpm (about 1600*g*), 10 min]. Dipotassium ethylenediaminetetraacetic acid was used as an anticoagulant. The plasma obtained was frozen at ≤ − 20 °C until analysis.

The primary PK endpoints were the volume of distribution (*V*_z_), clearance (CL), and biological half-life (*t*_½_) for thiotepa following the initial dose. Secondary PK endpoints were terminal phase elimination constant (*λ*_z_) and area under the plasma concentration curve from treatment initiation (0) through a specific time (AUC_0−*t*_) and through infinity (AUC_0−∞_), following the initial thiotepa dose. Additionally, the abovementioned parameters were calculated for TEPA as secondary endpoints.

*V*_z_ was calculated as $V_{\text{z}} = \frac{\text{CL }}{{\lambda_{\text{z}} }}$. For pediatric patients (24-h infusion), CL was calculated as ${\text{CL}} = \frac{{R_{ \inf } }}{{C_{\text{ss}} }}$ where Rinf is the infusion rate and *C*_ss_ is the concentration at the end of infusion. For adult patients (2-h infusion) CL was calculated as ${\text{CL}} = \frac{{R_{ \inf } }}{{C_{\text{p}} }} \times \left( {1 - {\text{e}}^{{ - \lambda_{\text{z}} \times t}}  } \right)$ where *C*_p_ is the concentration at the end of infusion and *t* is the end of infusion time. *λ*_z_ was calculated by linear regression of the terminal points of the log-linear concentration–time curve and *t*_½_ was determined as ln2/*λ*_z_. AUC_0–*t*_ was calculated by linear up/log down trapezoidal summation from the start of infusion (time zero) to *t*, *t* = 30 h for pediatric patients (6 h after a 24-h infusion), *t* = 10 h for adult patients (8 h after a 2-h infusion). AUC_0−∞_ was calculated as the following equation: AUC_0–last_ + *C*_last_/*λ*_z_. These calculations were conducted by IQVIA services Japan (Tokyo, Japan) using Phoenix^®^ WinNonlin^®^ version 6.4 or higher (Certara, LP, New Jersey, USA) and SAS version 9.4 or higher (SAS Institute, North Carolina, USA).

#### Analytical methods for assessing plasma concentration

Plasma thiotepa and its metabolite TEPA concentrations were determined by a validated liquid chromatography–tandem mass spectrometry (LC–MS/MS) method. To determine thiotepa and TEPA concentrations in human plasma samples, thiotepa, TEPA, and internal standard (hexamethylphosphoramide) were extracted from 50 μL of human plasma by protein precipitation using acetonitrile/methanol solution (1:1, v/v). Thiotepa, TEPA, and IS were separated by reverse-phase liquid chromatography (flow rate: 0.4 mL/min) using a C18 column with gradient elution (10 mmol/L ammonium formate solution: methanol, 9:1 for 0–1 min then linear gradient to 6:4 for 1–3 min, keep 6:4 for 3–5.5 min, then switch to 0:10 for 5.5–7 min, re-conditioning 9:1 for 7–9 min). The precursor > product ion transition for thiotepa (*m*/*z* 190 > 147), TEPA (*m*/*z* 174 > 131), and IS (*m*/*z* 180 > 135) were monitored in positive, electrospray ionization, multiple reaction mode.

The quantification ranges for thiotepa and TEPA in plasma were both 5–2500 ng/mL. The calibration curves of thiotepa and TEPA constructed using the 1/*X*^2^ weighting factor showed good linearity. Accuracy values for thiotepa and TEPA was − 1.8 to 2.0% and − 3.2 to 4.4%, respectively.

In the within-run accuracy and precision test for thiotepa at 5, 10, 200, and 2000 ng/mL, the precision was 8.8%, 4.3%, 2.1%, and 0.5%, and the accuracy of mean was − 2.6%, − 6.3%, − 4.0%, and − 4.0%, respectively. For TEPA at 5, 10, 200, and 2000 ng/mL, the precision was 7.9%, 4.7%, 2.3%, and 2.3%, and the accuracy of mean was − 14.4%, − 14.2%, − 12.0%, and − 13.5%, respectively. In the between-run accuracy and precision test for thiotepa at 5, 10, 200, and 2000 ng/mL, the precision was 6.6%, 6.3%, 3.5%, and 2.5%, and the accuracy of mean was 0.6%, − 2.5%, 0.5%, and − 0.5%, respectively. For TEPA at 5, 10, 200, and 2000 ng/mL, the precision was 9.9%, 7.1%, 7.3%, and 7.4%, and the accuracy of mean was − 7.0%, − 7.4%, − 3.5%, and − 5.0%, respectively.

Thiotepa and TEPA in processed sample were stable in an autosampler at 4 °C for 97 h and in human plasma at room temperature for 24 h. Thiotepa in human plasma was stable at − 20 °C for 61 days and after 5 freeze–thaw cycles. TEPA in human plasma was stable at − 20 °C for 17 days and after 3 freeze–thaw cycles.

Analysis of the plasma concentration of thiotepa and TEPA was conducted by Shin Nippon Biomedical Laboratories, Ltd., Tokyo, Japan. Analyses were carried out using the LC-10A liquid chromatography system (Shimadzu Co., Kyoto, Japan). Separations were conducted by using a YMC-Triart C18 (2.1 mm I.D. × 100 mm, 3 μm; YMC Co., Ltd., Kyoto, Japan). Detection was performed with an API4000 quadrupole mass spectrometry system (AB Sciex, Framingham, USA), equipped with turbo ion spray interface, operated in the positive mode, and configured in multiple reaction monitoring mode. The mass spectrometry system was operated using Analyst 1.6.1 software (AB Sciex, Framingham, USA). Thiotepa analytical standard was purchased from US Pharmacopeial Convention, Rockville, USA. TEPA analytical standard was purchased from Toronto Research Chemicals Inc., Toronto, Canada. Internal standard, hexamethylphosphoramide was purchased from Sigma-Aldrich Co. LLC, St. Louis, USA. High-performance liquid chromatography grade or special grade were supplied for other regents. High-purity deionized water was obtained from Milli-Q Advantage A10 (Merck KGaA, Darmstadt, Germany).

#### Safety

AEs and serious AEs were recorded from treatment initiation through to Day 28 post-HSCT. Clinical laboratory tests (hematology, blood chemistry, urine analysis), vital signs and ECOG performance status were recorded at baseline and HSCT Day − 7 (pediatric patients with solid tumors or brain tumors) or Day − 4 (adult patients with malignant lymphoma) and Day − 1, and on Days 7, 14, 21, and 28 post-HSCT. eGFR was recorded at baseline and Day 7 (pediatric patients with solid tumors or brain tumors only) and Day 28 post-HSCT. In addition, 12-lead electrocardiography (ECG) was performed at baseline and HSCT Day − 7 (pediatric patients with solid tumors or brain tumors) or Day − 4 and Day − 1 (adult patients with malignant lymphoma), and Days 7 and 28 post-HSCT. Echocardiography was performed to measure the left ventricular ejection fraction at baseline and HSCT Days 7 and 28. All safety assessments were recorded at discontinuation unless precluded by the patient’s condition. Baseline safety assessments were made at the pre-registration screening visit (14 days prior to registration) and/or the baseline screening visit (7 days prior to treatment initiation); results of the assessments closest to the treatment initiation date were adopted as the baseline values.

#### Efficacy

Preliminary efficacy endpoints were bone marrow suppression rate (the proportion of subjects receiving thiotepa with neutrophil count < 500 mm^3^ at least once during the conditioning or 28-day post-HSCT period), engraftment rate (the proportion of subjects with engraftment [neutrophil count ≥ 500/mm^3^ on 3 consecutive days following bone marrow suppression and HSCT]), time to engraftment (for patients with evidence of engraftment), and survival rate at Day 100 post-HSCT [proportion of surviving patients with a two-sided 95% confidence interval (CI) for the estimate provided by the Kaplan–Meier method].

### Statistical analysis

There was no formal sample size calculation. PK data were analyzed in the PK analysis population (patients who received at least one dose of thiotepa and had blood concentration data available). Safety and efficacy were analyzed in the safety analysis population and the efficacy analysis population, respectively (both defined as patients who received at least a single dose of thiotepa). PK parameters were calculated and summary statistics presented by target disease. AEs were summarized by Medical Dictionary for Regulatory Activities version 19.1 System Organ Class and Preferred Term and tabulated by target disease.

## Results

### Patient demographics

Overall, 19 patients were enrolled in the study, including four pediatric patients with solid tumors (one patient each with the onset of retinoblastoma, Ewing’s sarcoma, or neuroblastoma, and one patient with a relapsed rhabdoid tumor of the kidney); five pediatric patients with brain tumors (two patients with onset and one with relapsed medulloblastoma and two with onset of atypical teratoid rhabdoid tumors); nine adult patients with primary CNS lymphoma (three with onset and six with relapse) and one adult patient with the onset of diffuse large cell B cell lymphoma with CNS involvement. No patients experienced multiple primary cancers. Patient demographics and characteristics are presented in Table 1 and Supplementary Table S1.

**Table 1:** Patient demographics (safety analysis population)

	Pediatric solid tumor or pediatric brain tumor *N* = 9	Adult malignant lymphoma *N* = 10
Sex		
Female, *n* (%)	4 (44.4)	4 (40.0)
Male, *n* (%)	5 (55.6)	6 (60.0)
Age, years		
Mean (SD)	7.0 (5.9)	53.7 (11.6)
Median (min, max)	5.0 (2, 16)	54.5 (35, 68)
Height, cm, mean (SD)	114.3 (34.4)	164.7 (10.2)
Weight, kg, mean (SD)	23.0 (14.4)	60.0 (12.1)
Body surface area,^a^ m^2^, mean (SD)	0.85 (0.40)	1.65 (0.20)
ECOG performance status,^b^*n* (%)		
0	4 (44.4)	7 (70.0)
1	5 (55.6)	3 (30.0)
Primary malignancy, *n* (%)		
Pediatric solid tumor	4 (44.4)	0
Pediatric brain tumor	5 (55.6)	0
Malignant lymphoma	0	10 (100.0)
Relapse, *n* (%)	2 (22.2)	6 (60.0)
Number of previous HSCTs, *n* (%)		
0	6 (66.7)	10 (100.0)
1	3 (33.3)	0
Patients with complications^c^	6 (66.7)	9 (90.0)

*ECOG* Eastern Cooperative Oncology Group, *HSCT* hematopoietic stem cell transplantation, *SD* standard deviation

^a^Body surface area is calculated as a function of age. If age was < 16 years, the Mosteller formula was used ([weight (kg) × height (cm)/3600]^0.5^). If age was ≥ 16 years, the DuBois formula was used ([weight (kg)^0.425^ × height (cm)^0.725^] × 0.007184)

^b^Range 0–4

^c^See Supplementary Table S1 for full list of complications

### Pharmacokinetics

**Fig. 1:**
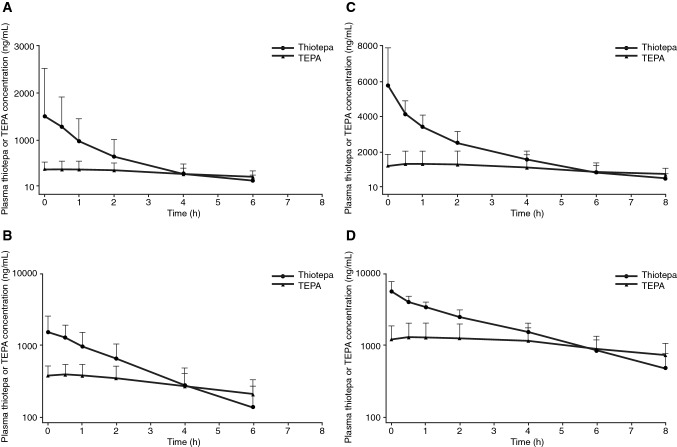
Plasma thiotepa and TEPA metabolite concentration following initial 24-h thiotepa IV infusion in pediatric patients with solid tumors or brain tumors on a linear (**a**) and semi-logarithmic (**b**) scale and following the initial 2-h thiotepa IV infusion in adult patients with malignant lymphoma on a linear (**c**) and semi-logarithmic (**d**) scale (pharmacokinetic analysis population). *TEPA* triethylene phosphoramide

**Fig. 2:**
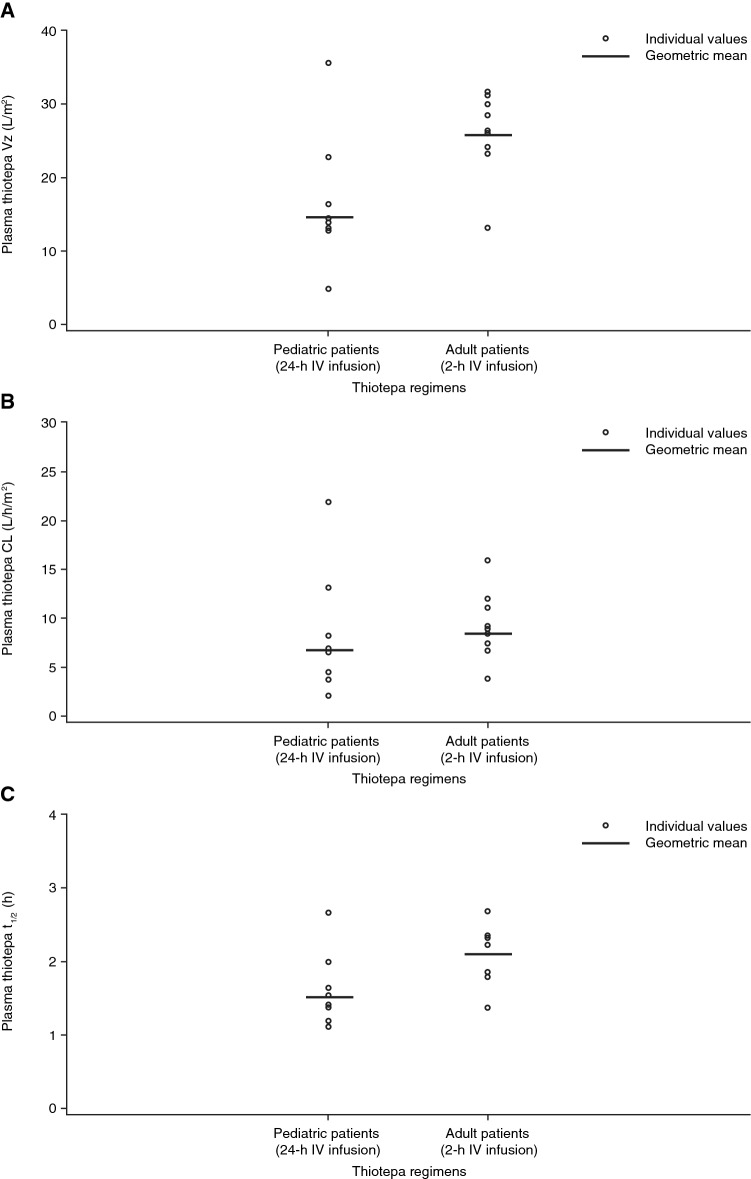
Scatterplot of *V*_z_ (**a**), CL (**b**), and *t*_½_ (**c**) following initial 24-h thiotepa IV infusion in pediatric patients with solid tumors or brain tumors and 2-h thiotepa IV infusion in patients with malignant lymphoma (pharmacokinetic analysis population). *CL* clearance, *IV* intravenous, *t*_*½*_ biological half-life, *V*_*z*_ volume of distribution

Plasma concentration curves for thiotepa and its metabolite, TEPA, are presented in Fig. 1a, b for pediatric patients with solid tumors or brain tumors and Fig. 1c, d for adult patients with malignant lymphoma. For both sets of patients, TEPA had a lower peak concentration and demonstrated a slower elimination rate compared with thiotepa. Scatterplots of individual values of the primary PK parameters *V*_z_, CL, and *t*_½_ for thiotepa are presented in Fig. 2a–c, respectively. For pediatric patients with solid tumors or brain tumors, the arithmetic mean (coefficient of variation) for *V*_z_, CL, and *t*_½_ for thiotepa was 16.4 L/m^2^ (51.9%), 8.2 L/h/m^2^ (73.5%), and 1.6 h (31.4%), respectively (Supplementary Table S2). For patients with malignant lymphoma, these values were 26.4 L/m^2^ (21.0%), 9.0 L/h/m^2^ (37.4%), and 2.1 h (19.7%), respectively (Supplementary Table S2). Secondary PK endpoints are also shown in Supplementary Table S2.

### Safety

All 19 patients in the study experienced at least one treatment-emergent AE (TEAE) during the reporting period (from treatment initiation to 28 days post HSCT). TEAEs possibly, probably, or definitely related to thiotepa are shown in Table 2. There were no TEAEs leading to discontinuation or suspension of thiotepa. Two serious AEs (Grade 4 and 5) occurred during the reporting period in pediatric patients with solid tumors or brain tumors and a causal relationship with thiotepa could not be excluded. A male patient of 2 years of age with rhabdoid tumor of the kidney and previous HSCT (14 months prior to study) developed cardiac arrest 2 days post-HSCT and died 10 days later. A female patient of 17 years of age (16 years of age at study registration) with medulloblastoma and previous HSCT (5 months prior to study) developed pulmonary edema on Day 12 post-HSCT. This patient later developed mediastinal emphysema and interstitial pneumonia triggered by systemic herpes zoster and died due to the progression of pulmonary fibrosis on Day 78 post-HSCT.

**Table 2:** TEAEs related^a^ to thiotepa occurring in ≥ 2 patients in either target disease group by system organ class and preferred term (safety analysis population)

System organ class Preferred term, *n* (%)	Pediatric solid tumor or pediatric brain tumor *N* = 9	Adult malignant lymphoma *N* = 10	Total *N* = 19
Blood and lymphatic system disorders	5 (55.6)	10 (100.0)	15 (78.9)
Febrile neutropenia	5 (55.6)	10 (100.0)	15 (78.9)
Cardiac disorders	3 (33.3)	0	3 (15.8)
Pericardial effusion	2 (22.2)	0	2 (10.5)
Gastrointestinal disorders	9 (100.0)	10 (100.0)	19 (100.0)
Diarrhea	8 (88.9)	8 (80.0)	16 (84.2)
Nausea	6 (66.7)	10 (100.0)	16 (84.2)
Stomatitis	9 (100.0)	5 (50.0)	14 (73.7)
Vomiting	9 (100.0)	4 (40.0)	13 (78.9)
Oral disorder	0	4 (40.0)	4 (21.1)
Abdominal pain	2 (22.2)	0	2 (10.5)
Anal inflammation	2 (22.2)	0	2 (10.5)
Enterocolitis	2 (22.2)	0	2 (10.5)
Proctalgia	0	2 (20.0)	2 (10.5)
General disorders and administration site conditions	6 (66.7)	2 (20.0)	8 (42.1)
Malaise	3 (33.3)	1 (10.0)	4 (21.1)
Face edema	3 (33.3)	0	3 (15.8)
Pyrexia	2 (22.2)	1 (10.0)	3 (15.8)
Edema peripheral	2 (22.2)	0	2 (10.5)
Infections and infestations	3 (33.3)	1 (10.0)	4 (21.1)
Bacterial infection	3 (33.3)	0	3 (15.8)
Investigations	7 (77.8)	7 (70.0)	14 (73.7)
ALT increased	6 (66.7)	6 (60.0)	12 (63.2)
AST increased	6 (66.7)	4 (40.0)	10 (52.6)
GGT increased	3 (33.3)	4 (40.0)	7 (36.8)
Blood alkaline phosphatase increased	0	2 (20.0)	2 (10.5)
Metabolism and nutrition disorders	9 (100.0)	8 (80.0)	17 (89.5)
Decreased appetite	7 (77.8)	7 (70.0)	14 (73.7)
Hypoalbuminemia	6 (66.7)	0	6 (31.6)
Hypokalemia	1 (11.1)	2 (20.0)	3 (15.8)
Hypocalcemia	2 (22.2)	0	2 (10.5)
Nervous system disorders	5 (55.6)	6 (60.0)	11 (57.9)
Dysgeusia	2 (22.2)	6 (60.0)	8 (42.1)
Psychiatric disorders	2 (22.2)	1 (10.0)	3 (15.8)
Anxiety	2 (22.2)	0	2 (10.5)
Renal and urinary disorders	4 (44.4)	0	4 (21.1)
Acute kidney injury	2 (22.2)	0	2 (10.5)
Respiratory, thoracic and mediastinal disorders	3 (33.3)	1 (10.0)	4 (21.1)
Pleural effusion	2 (22.2)	0	2 (10.5)
Pulmonary edema	2 (22.2)	0	2 (10.5)
Skin and subcutaneous tissue disorders	8 (88.9)	4 (40.0)	12 (63.2)
Alopecia	1 (11.1)	3 (30.0)	4 (21.1)
Rash maculopapular	2 (22.2)	2 (20.0)	4 (21.1)
Skin hyperpigmentation	4 (44.4)	0	4 (21.1)
Dry skin	2 (22.2)	0	2 (10.5)
Vascular disorders	2 (22.2)	0	2 (10.5)
Capillary leak syndrome	1 (11.1)	0	1 (5.3)
Hypertension	1 (11.1)	0	1 (5.3)

TEAEs were defined as AEs which started on the day of or any day after treatment initiation with thiotepa, up to Day 28 post-HSCT. All AEs were coded using MedDRA dictionary version 19.1. Multiple reports of AEs that map to a common System Organ Class and Preferred Term counted only once for each patient

*AE* adverse event, *ALT* alanine aminotransferase, *AST* aspartate aminotransferase, *GTT* γ-glutamyltransferase, *MedDRA* Medical Dictionary for Regulatory Activities, *TEAE* treatment-emergent AE

^a^TEAEs with a causal relationship determined by the investigator as ‘possibly’, ‘probably’, or ‘definitely’ related to thiotepa

There were no clinically significant changes in vital signs, body weight, 12-lead ECG, or left ventricular ejection fraction in pediatric patients with solid tumors or brain tumors or adult patients with malignant lymphoma. Clinical laboratory values related to bone marrow suppression significantly decreased after treatment initiation but appeared recovered by Day 28 post-HSCT. Other than hematology tests, abnormalities of ≥ Grade 3 that occurred in ≥ 2 patients by target disease were γ-glutamyltransferase (three pediatric patients with solid or brain tumors and four patients with malignant lymphoma), potassium (three pediatric patients with solid tumor or brain tumors), and alanine aminotransferase (three patients with malignant lymphoma).

### Efficacy

Briefly, for pediatric patients with solid tumors or brain tumors, bone marrow suppression rate was 100% and engraftment rate was 66.7% (6/9 patients). For patients with engraftment, mean time to engraftment was 14.8 days. Survival rate at Day 100 post-HSCT was 77.8% (95% CI 36.5–93.9%). For patients with lymphoma, bone marrow suppression rate and engraftment rate were 100%. Mean time to engraftment was 11.3 days. Survival rate at Day 100 post-HSCT was 100%.

## Discussion

Thiotepa has been used as a chemotherapeutic agent for more than 70 years [1, 2]. While numerous studies have examined the PK of thiotepa [10, 20–31], to our knowledge, this is the first such study in a Japanese population.

Standard conditioning regimens have not been defined for HSCT [32]. Regimens have largely been evaluated in Phase 1 and 2 trials and there is a paucity of Phase 3 data [32]. However, thiotepa has been used as a conditioning agent for HSCT since the 1980s, in a range of malignancies and in combination with a variety of agents [5, 6, 11, 20, 33–37], and in 2010 its use as a conditioning agent for HSCT in hematological diseases and solid tumors was approved in Europe based on ‘well-established medicinal use’ [17, 38]. Thiotepa is used as a conditioning agent for HSCT in CNS tumors due to its effective penetration of the cerebrospinal fluid [10, 36, 39]. A meta-analysis of the use of thiotepa in combination with other agents prior to HSCT in patients with CNS lymphoma estimated that 75.9% of patients achieved complete remission and 61.7% had progression-free survival up to 125 months following treatment, although none of the studies included in the analysis had a priori controls [36]; 25.5% of patients experienced relapse, 24.6% experienced infection, and 13.2% experienced neurotoxicity [36]. In the present study, in pediatric patients with solid tumors or brain tumors, a double conditioning regimen comprising two cycles of thiotepa separated by 1 week was employed, similar to that used by Hara and colleagues in Japanese patients with pediatric solid tumors [8]. Such a regimen is thought to allow administration of near maximal doses of thiotepa and melphalan, whilst reducing the incidence of toxicities such as mucositis and neurotoxicity, compared with continuous regimens [8].

The most common TEAEs were diarrhea and nausea (each 84.2%), vomiting (78.9%), stomatitis, and reduced appetite (each 73.7%) and elevated aminotransferases (52.6–63.2%). These TEAEs are consistent with the known safety profile of thiotepa [17]. Vomiting observed before transplantation was not common in the patients who received aprepitant or fosaprepitant (2/6; 33.3%). Thiotepa conditioning prior to autologous HSCT, with careful monitoring of patient condition, was considered to be well tolerated in this study.

As busulfan may induce convulsion, prophylactic anti-convulsants were recommended; 2 of 9 children received levetricetam (IV or PO) and 5 of 11 adults received valproate sodium (PO). The major metabolic pathway of levetiracetam is the enzymatic hydrolysis of the acetamide group, and no CYP inhibition or induction. [40]. Levetiracetam has less possibility of drug–drug interaction (DDI) on metabolism with thiotepa, busulfan, and melphalan. The major metabolic pathways of valproate are mitochondrial β-oxidation, microsomal ω- and (ω − 1)-hydroxylation, and glucuronidation [41]. Valproate has less possibility of DDI on metabolism with thiotepa, busulfan, and melphalan. However, valproate DDI was reported on protein-binding so it could not be assured that there was no interaction with thiotepa, busulfan, or melphalan in this study. [42].

Thiotepa is primarily metabolized by the hepatic system, where it is rapidly biotransformed by CYP3A4 and CYP2B6 to its key metabolite, TEPA, which is also an alkylating agent [3, 17]. The plasma–concentration curves in pediatric patients with solid tumors or brain tumors, and malignant lymphoma showed a rapid decline in thiotepa concentration following IV administration, with a slower elimination phase for the TEPA metabolite, as expected. In an earlier study, TEPA showed a slower elimination rate than thiotepa [23]. In addition, thiotepa is an alkylating agent with a strong chemical reactivity and a very short half-life.

The mean volume of distribution (*V*_z_) of thiotepa normalized with body surface area (BSA) was lower for pediatric patients (16.4 L/m^2^) compared with adult patients (26.4 L/m^2^) as expected due to the higher specific surface area of children. There were no marked differences in thiotepa elimination between pediatric patients with solid tumors or brain tumors and adult patients with malignant lymphoma. The *t*_½_ for thiotepa was 1.6 h in pediatric patients with solid tumors or brain tumors and 2.1 h in adult patients with malignant lymphoma, whilst for TEPA it was 6.1 and 6.7 h, respectively. Thiotepa clearance was 8.2 L/h/m^2^ in pediatric patients with solid tumors and brain tumors and 9.0 L/h/m^2^ in adult patients with malignant lymphoma.

It is difficult to compare studies directly because of differences in drug administration, measurement of concentration, and calculations of PK parameters; however, similar studies have been done evaluating thiotepa in pediatric patients by Heideman et al. and in adult patients by O’Dwyer et al. [10, 29].

Patient baseline characteristics have previously been identified to influence the PK of thiotepa and TEPA. For example, Huitema et al. found that thiotepa clearance was related to serum alkaline phosphatase concentrations, while the volume of distribution was related to serum total protein concentration and elimination of TEPA was related to body weight [25]. Ekhart et al. found that polymorphisms of drug-metabolizing enzymes glutathione *S*-transferase, CYP2B6, and CYP3A affect the PK of thiotepa and TEPA [24]. The PK of some medicines are known to be influenced by race or ethnicity [43, 44]. It is, therefore, important to investigate PK of thiotepa in different populations, particularly given that changes in exposure are known to affect its safety profile, with increased exposure to thiotepa associated with heightened transaminase levels and greater exposure to TEPA related to increased mucositis [26].

It was difficult to discuss the DDIs on thiotepa PK with busulfan and melphalan in this study because of a lack of PK data on thiotepa with and without concomitant drugs in the same patient. According to previous reports, there is little possibility of major DDI on the metabolism of thiotepa, busulfan and melphalan; thiotepa is metabolized mainly by CYP2B6 and 3A4 [3]; busulfan by glutathione *S*-transferase [45]; and melphalan by non-enzymatic reaction. [46] There were few reports about CYP2B6 and 3A4 inhibition by busulfan or melphalan; however, it was reported that busulfan did not inhibit CYP3A4 and might accelerate metabolism by CYP2A6 and 2E1 [47]. However, DDI on distribution and excretion were unknown and further information is needed to discuss DDI.

## Conclusion

There is unmet clinical need for thiotepa in Japan [48]. Whilst the safety and efficacy of thiotepa have been established over 70 years of use, to our knowledge, this is the first time the PK have been assessed in a Japanese population. The PK of thiotepa and TEPA were similar between pediatric patients with solid or brain tumors and adult patients with malignant lymphoma, with the exception of *V*_z_, which, as expected, was greater in adults.

## Electronic supplementary material

Below is the link to the electronic supplementary material.

**Supplementary Fig. S1** Clinical study flowchart. ▼ N, denotes the number of times that blood samples were collected on HSCT Day. HSCT, HSCT, hematopoietic stem cell transplantation; IC, informed consent; PK, pharmacokinetic (EPS 1719 kb)

Supplementary material 2 (DOCX 27 kb)

## Data Availability

The datasets generated during and/or analyzed during the current study are available from the corresponding author on reasonable request.
