# Supplementary material for: Pharmacokinetics of thiotepa in high-dose regimens for autologous hematopoietic stem cell transplant in Japanese patients with pediatric tumors or adult lymphoma
Source: Cancer Chemother Pharmacol. 2019 Aug 19;84(4):849–60. doi: 10.1007/s00280-019-03914-2 (PMC6768914; doi:10.1007/s00280-019-03914-2)
Supplement: Supplementary file 2 — Supplementary material 2 (DOCX 27 kb) [file 280_2019_3914_MOESM2_ESM.docx]

**Supplementary Table S1** Patient baseline complications (safety analysis population)

| **System Organ Class, n (%)**  **Preferred term, n (%)** | **Pediatric  solid tumor or pediatric brain tumor N=9** | **Adult malignant lymphoma N=10** | **Total N=19** |
| --- | --- | --- | --- |
| Subjects with any complication | 6 (66.7) | 9 (90.0) | 15 (78.9) |
| Blood and lymphatic system disorders | 2 (22.2) | 0 | 2 (10.5) |
| Anemia | 2 (22.2) | 0 | 2 (10.5) |
| Endocrine disorders | 2 (22.2) | 0 | 2 (10.5) |
| Adrenal insufficiency | 1 (11.1) | 0 | 1 (5.3) |
| Diabetes insipidus | 1 (11.1) | 0 | 1 (5.3) |
| Hypopituitarism | 1 (11.1) | 0 | 1 (5.3) |
| Eye disorders | 1 (11.1) | 3 (30.0) | 4 (21.1) |
| Dry eye | 0 | 2 (20.0) | 2 (10.5) |
| Glaucoma | 0 | 1 (10.0) | 1 (5.3) |
| Strabismus | 1 (11.1) | 0 | 1 (5.3) |
| Gastrointestinal disorders | 1 (11.1) | 4 (40.0) | 5 (26.3) |
| Constipation | 1 (11.1) | 2 (20.0) | 3 (15.8) |
| Stomatitis | 0 | 2 (20.0) | 2 (10.5) |
| Hemorrhoids | 0 | 1 (10.0) | 1 (5.3) |
| Proctalgia | 0 | 1 (10.0) | 1 (5.3) |
| General disorders and administration site conditions | 1 (11.1) | 0 | 1 (5.3) |
| Face edema | 1 (11.1) | 0 | 1 (5.3) |
| Edema peripheral | 1 (11.1) | 0 | 1 (5.3) |
| Immune system disorders | 0 | 4 (40.0) | 4 (21.1) |
| Seasonal allergy | 0 | 4 (40.0) | 4 (21.1) |
| Infections and infestations | 1 (11.1) | 0 | 1 (5.3) |
| Chronic sinusitis | 1 (11.1) | 0 | 1 (5.3) |
| Injury, poisoning, and procedural complications | 1 (11.1) | 0 | 1 (5.3) |
| Radiation skin injury | 1 (11.1) | 0 | 1 (5.3) |
| Investigations | 2 (22.2) | 0 | 2 (10.5) |
| Platelet count decreased | 2 (22.2) | 0 | 2 (10.5) |
| Lymphocyte count decreased | 1 (11.1) | 0 | 1 (5.3) |
| Neutrophil count decreased | 1 (11.1) | 0 | 1 (5.3) |
| White blood cell count decreased | 1 (11.1) | 0 | 1 (5.3) |
| Musculoskeletal and connective tissue disorders | 0 | 1 (10.0) | 1 (5.3) |
| Neck pain | 0 | 1 (10.0) | 1 (5.3) |
| Nervous system disorders | 1 (11.1) | 4 (40.0) | 5 (26.3) |
| Neuropathy peripheral | 0 | 4 (40.0) | 4 (21.1) |
| Monoplegia | 1 (11.1) | 0 | 1 (5.3) |
| Neuralgia | 1 (11.1) | 0 | 1 (5.3) |
| Psychiatric disorders | 0 | 5 (50.0) | 5 (26.3) |
| Insomnia | 0 | 5 (50.0) | 5 (26.3) |
| Respiratory, thoracic, and mediastinal disorders | 2 (22.2) | 1 (10.0) | 3 (15.8) |
| Rhinitis, allergic | 1 (11.1) | 1 (10.0) | 2 (10.5) |
| Asthma | 1 (11.1) | 0 | 1 (5.3) |
| Skin and subcutaneous tissue disorders | 1 (11.1) | 0 | 1 (5.3) |
| Urticaria | 1 (11.1) | 0 | 1 (5.3) |
| Vascular disorders | 0 | 4 (40.0) | 4 (21.1) |
| Hypertension | 0 | 4 (40.0) | 4 (21.1) |
| Subclavian vein thrombosis | 0 | 1 (10.0) | 1 (5.3) |

**Supplementary Table S2.** Pharmacokinetic parameters following the initial thiotepa infusion in pediatric patients with solid tumors or brain tumors or adult patients with malignant lymphoma, calculated from plasma thiotepa or TEPA metabolite concentration (pharmacokinetic analysis population)

|  |  | **V_z_  (L/m^2^)** | **CL  (L/h/m^2^)** | **t_½_  (h)** | **AUC_0-t_  (h*ng/mL)^a^** | **AUC_0-∞_ (h*ng/mL)** | **λ_z_  (/h)** |
| --- | --- | --- | --- | --- | --- | --- | --- |
| **Pediatric solid tumors or brain tumors 24-hour thiotepa infusion** | | | | | | | |
| Thiotepa | n | 9 | 9 | 9 | 9 | 9 | 9 |
|  | Geometric mean | 14.6 | 6.7 | 1.5 | 17820.1 | 18071.6 | 0.5 |
|  | Geometric CV (%) | 57.6 | 77.2 | 28.6 | 77.0 | 78.2 | 28.6 |
|  | Median | 14.2 | 6.9 | 1.4 | 17584.7 | 17774.2 | 0.5 |
|  | Minimum | 4.8 | 2.1 | 1.1 | 5395 | 5416 | 0.3 |
|  | Maximum | 35.6 | 21.9 | 2.7 | 51707 | 52318 | 0.6 |
|  | Arithmetic mean | 16.4 | 8.2 | 1.6 | 21616.9 | 22013.4 | 0.5 |
|  | SD | 8.5 | 6.0 | 0.5 | 14120.6 | 14456.5 | 0.1 |
|  | CV (%) | 51.9 | 73.5 | 31.4 | 65.3 | 65.7 | 25.1 |
| TEPA | n | 9 | 9 | 9 | 9 | 9 | 9 |
|  | Geometric mean | 207.8 | 27.1 | 5.3 | 5707.8 | 7370.1 | 0.1 |
|  | Geometric CV (%) | 63.1 | 74.8 | 55.2 | 62.5 | 74.8 | 55.2 |
|  | Median | 176.9 | 27.3 | 4.5 | 6198.3 | 7314.9 | 0.2 |
|  | Minimum | 116.4 | 10.2 | 2.8 | 1480 | 1656 | 0.0 |
|  | Maximum | 657.1 | 120.8 | 15.6 | 9428 | 19605 | 0.2 |
|  | Arithmetic mean | 247.3 | 34.6 | 6.1 | 6381.0 | 8677.7 | 0.1 |
|  | SD | 180.6 | 33.1 | 4.0 | 2520.6 | 4899.8 | 0.1 |
|  | CV (%) | 73.0 | 95.7 | 65.2 | 39.5 | 56.5 | 41.5 |
| **Adult malignant lymphoma, 2-hour thiotepa infusion** | | | | | | | |
| Thiotepa | n | 10 | 10 | 10 | 10 | 10 | 10 |
|  | Geometric mean | 25.7 | 8.5 | 2.1 | 20621.1 | 22084.0 | 0.3 |
|  | Geometric CV (%) | 26.7 | 40.6 | 21.2 | 24.7 | 27.4 | 21.2 |
|  | Median | 27.4 | 8.6 | 2.3 | 20913.8 | 22521.1 | 0.3 |
|  | Minimum | 13.0 | 3.8 | 1.4 | 14338 | 14843 | 0.3 |
|  | Maximum | 31.7 | 16.0 | 2.7 | 30143 | 32363 | 0.5 |
|  | Arithmetic mean | 26.4 | 9.0 | 2.2 | 21177.1 | 22807.0 | 0.3 |
|  | SD | 5.5 | 3.4 | 0.4 | 5159.3 | 6052.8 | 0.1 |
|  | CV (%) | 21.0 | 37.4 | 19.7 | 24.4 | 26.5 | 22.4 |
| TEPA | n | 10 | 10 | 10 | 10 | 10 | 10 |
|  | Geometric mean | 120.3 | 12.6 | 6.6 | 9132.9 | 15870.5 | 0.1 |
|  | Geometric CV (%) | 47.4 | 43.0 | 17.2 | 45.8 | 43.0 | 17.2 |
|  | Median | 131.0 | 13.5 | 6.5 | 8832.0 | 14999.4 | 0.1 |
|  | Minimum | 44.3 | 5.26 | 5.09 | 5389 | 8793 | 0.1 |
|  | Maximum | 195.3 | 22.7 | 8.80 | 23895 | 38040 | 0.1 |
|  | Arithmetic mean | 130.1 | 13.5 | 6.7 | 10059.5 | 17248.9 | 0.1 |
|  | SD | 47.4 | 5.0 | 1.2 | 5389.1 | 8303.7 | 0.0 |
|  | CV (%) | 36.5 | 36.9 | 17.3 | 53.6 | 48.1 | 16.9 |

^a^AUC_0–30_ for 24-hour IV infusion (in pediatric patients with solid tumors or brain tumors) and AUC_0–10_ for 2-hour infusion (in adult patients with malignant lymphoma)

λz, phase elimination constant; AUC_0–t_, area under the plasma concentration curve from treatment initiation through time; AUC_0–∞_, area under the plasma concentration curve from treatment initiation through infinity; CL, clearance; t_½_, biological half-life; CV, coefficient of variation; SD, standard deviation; TEPA, triethylene phosphoramide; V_z_, mean volume of distribution
